# Emergency Retroperitoneal Sarcoma Surgery for Preoperative Rupture and Hemoperitoneum: A Case Report

**DOI:** 10.7759/cureus.13936

**Published:** 2021-03-17

**Authors:** Laura Samà, Dimitri Tzanis, Toufik Bouhadiba, Sylvie Bonvalot

**Affiliations:** 1 Sarcoma, Melanoma and Rare Tumors Surgery Unit, Humanitas Clinical and Research Center - IRCCS, MIlan, ITA; 2 Department of Biomedical Sciences, Humanitas University, Milan, ITA; 3 Department of Surgery, Institut Curie, Paris Sciences et Lettres University, Paris, FRA; 4 Department of Surgery, Institut Curie, Paris Sciences et Lettres University University, Paris, FRA

**Keywords:** retroperitoneal sarcoma, rupture, perforation, hemoperitoneum, bleeding, sarcoma surgery, emergency

## Abstract

Retroperitoneal sarcoma (RPS) is a rare and heterogeneous tumor. A percutaneous core needle biopsy (PCNB) is required for obtaining a histological diagnosis of the condition and for planning the therapy. Surgery is the standard of care for RPS; it is a standardized procedure, and it should be performed in a referral sarcoma center. Sarcoma surgery is rarely performed on an urgent or emergent basis. In this report, we describe a case of a retroperitoneal leiomyosarcoma that presented with spontaneous rupture and hemoperitoneum, which required surgical treatment in an urgent manner. To our knowledge, this is the first case of hemoperitoneum due to RPS rupture to be reported in the literature.

## Introduction

Retroperitoneal sarcomas (RPS) is a rare tumor characterized by wide heterogeneity in histology and behavior. Its annual worldwide incidence is 0.76 new cases per 100,000 people according to the latest findings [1]. Usually, symptoms appear once the tumor becomes large enough and they are usually non-specific such as malnutrition, pain, abdominal distension, and gastrointestinal obstruction [2]. In cases of retroperitoneal mass, a preoperative percutaneous core needle biopsy (PCNB) is necessary to reach a correct histologic diagnosis before planning any therapy [3]. Surgery is the mainstay of treatment for RPS. The quality of the surgical procedure is crucial for ensuring a potential cure. Surgical positive margins, gross residual disease, tumor rupture, and high grade of the disease are associated with decreased overall survival (OS) and increased abdominal recurrence [4]. Local recurrence remains the leading cause of death in patients with RPS [5,6]. Hence, RPS surgery should be performed in a specialized referral sarcoma center. Surgery consists of the resection of the tumor en-bloc with adjacent viscera (involved or adherent) to optimize resection margins and to avoid tumor rupture [7,8]. In very rare cases, RPS surgery requires urgent management. We describe a case of left retroperitoneal leiomyosarcoma complicated by a spontaneous perforation and hemoperitoneum. Urgent surgery was the only possible life-saving treatment for the patient.

## Case presentation

**Figure 1:**
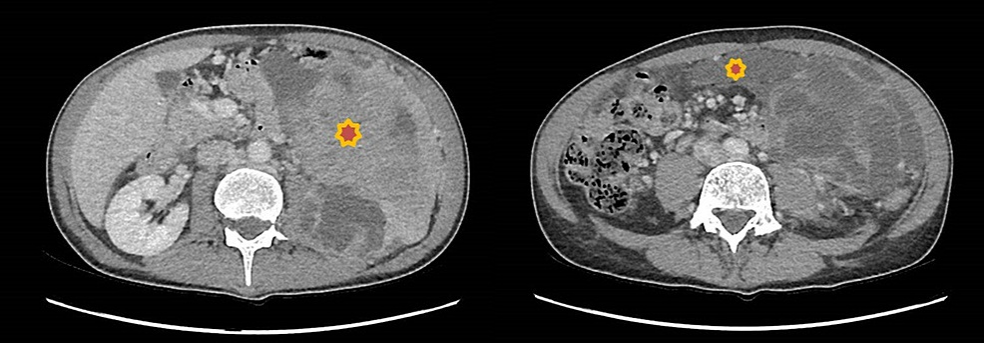
Preoperative abdominal CT scan The images show left retroperitoneal leiomyosarcoma (left) and hemoperitoneum (right) CT: computed tomography

A 53-year-old man presented to the emergency department of another hospital with abdominal pain and severe weight loss (12 kilograms). An abdominal CT scan showed a 10-cm left retroperitoneal mass. According to the latest European Society for Medical Oncology (ESMO) Guidelines [9], a CT-guided PCNB was performed. The diagnosis was as follows: leiomyosarcoma, of at least Fédération Nationale des Centres de Lutte Contre le Cancer (FNCLCC) grade 2 [10]. In light of the diagnosis of sarcoma and the appearance of sub-ileus symptoms with increased abdominal pain, the patient was transferred to our referral sarcoma center. A new CT scan was performed, which showed an increase in the volume of the left retroperitoneal necrotic mass (about 15 cm) and an intraperitoneal accumulation of fluid evoking a hemoperitoneum (Figure 1).

**Figure 2:**
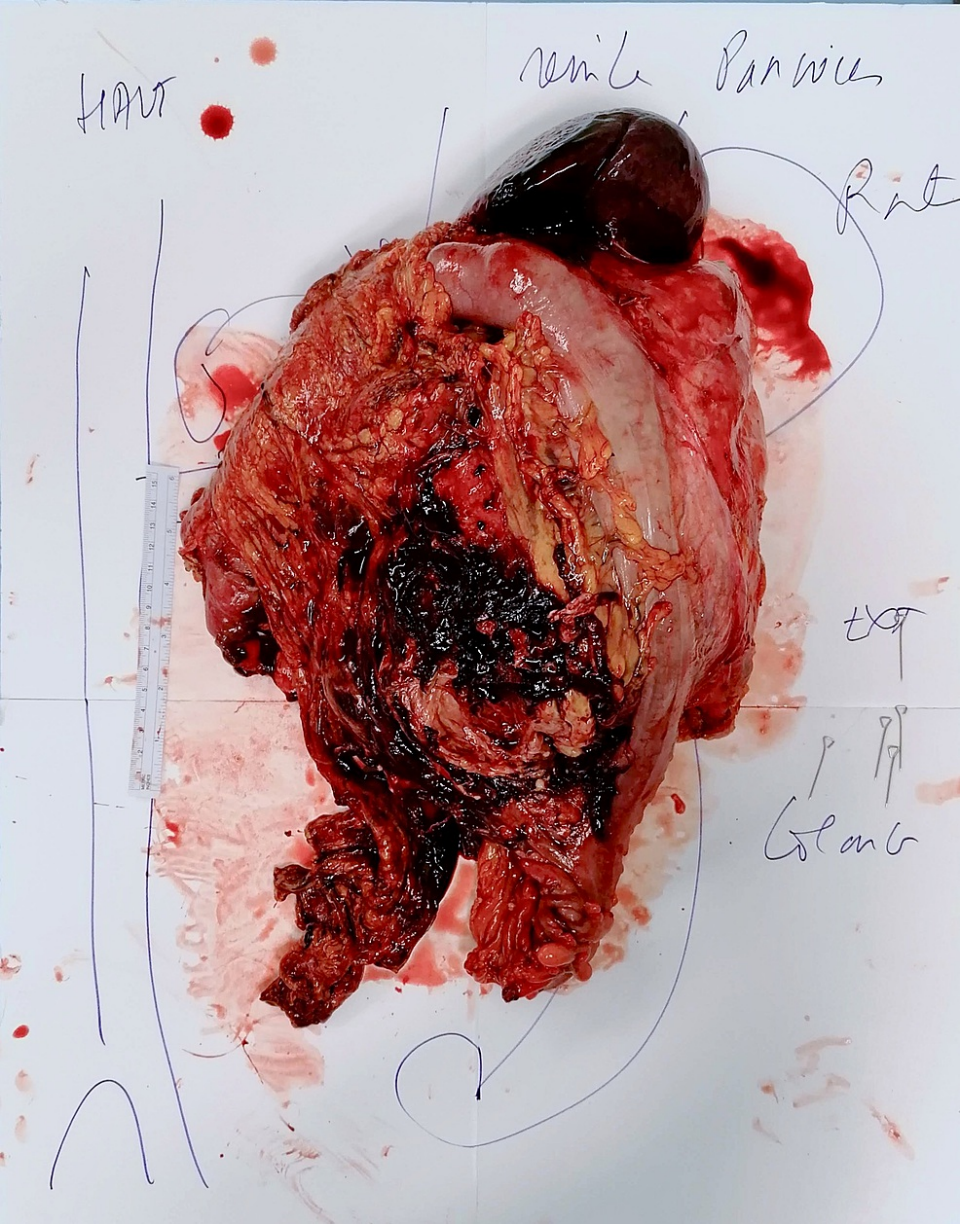
Removed specimen

The tumor was in direct contact with the left kidney, the pancreatic tail, and the psoas muscle. The superior mesenteric artery was free of infiltration. The chest CT scan was normal. Blood examination revealed severe malnutrition: hemoglobin of 9.6 g/dl, albumin of 24.8 g/l, and prealbumin 0.14 g/l. The coronavirus disease (COVID-19) nasal swab test was negative. Considering the clinical deterioration of the patient and the presence of hemoperitoneum, which proved the spontaneous bleeding of the mass, the decision to conduct surgery as a delayed emergency was made. A median laparotomy extending transversally to the left flank was performed, and 2.5 liters of hemorrhagic ascites were evacuated. A large tumor perforation was identified in the distal part of the tumor with colon perforation. The tumor had extensively invaded the peritoneal layer and it was spontaneously bleeding over the entire surface. The left kidney was completely encompassed by the tumor. The first step of surgical resection was the liberation of the root of the mesentery, contiguous but not infiltrated by the tumor. After coloepiploic separation, transverse colon and rectosigmoid junction were divided. The distal pancreas and spleen were indivisible from the tumor. Given the extension of the mass, we decided to perform a distal splenopancreatectomy. Vascular dissection was performed from the iliac vessels up to the aorta with a sub-adventitial dissection. In the left iliac fossa, the ureter was divided, and the femoral nerve was isolated. The left renal vein and artery were divided by stapler and ligation, respectively. The left psoas fascia was detached from the vertebra and it was left posteriorly to the tumor. Finally, the tumor was removed en-bloc with the left kidney, ipsilateral colon, mesocolon, spleen, and distal pancreas in front, aponeurosis of the psoas in the back, and parietal peritoneum on the side of the mass (Figure 2). The omentum was used to fill the surgical bed.

The postoperative course was characterized by intrabdominal bleeding due to a pancreatic fistula, which was treated at first by a splenic artery embolization on the 10th postoperative day (POD) and then by an exploratory laparotomy on the 14th POD. During surgery, no active bleeding had been found and peritoneal cavity lavage with saline solution had been performed. After the resolution of the pancreatic fistula, the surgical drains were removed and the patient was discharged on the 42nd POD. The histological exam revealed a 16-cm dedifferentiated leiomyosarcoma (FNCLCC grade 3). The tumor showed high mitotic activity (23 mitoses/10 high-power fields) and areas of necrosis (up to 50%). Resection margins were negative. Tumor rupture and colic perforation were confirmed by the pathologist. The multidisciplinary tumor board composed of surgical oncologists, oncologists, radiotherapists, radiologists, and pathologists discussed the clinical case after surgery and decided to have the patient undergo follow-up. A thoracoabdominal CT scan after four months was recommended. No further adjuvant systemic treatments were deemed to be indicated in this case.

## Discussion

Sarcoma surgery is an elective procedure, usually performed by an expert sarcoma team after PCNB. En-bloc resection with involved or adherent organs is a standard procedure with adjustments according to the presentation of sarcoma (location, size, and histology) [4,5]. In rare cases, RPS presents a life-threatening condition such as spontaneous perforation or bleeding. Data on RPS surgical approach in urgent situations are scarce in the literature. In our view, surgeons should have a firm grasp of the technical aspects of RPS surgery [5]. The morbidity rate related to PCNB is very low. In a recent series of 358 PCNBs in suspected RPS cases, seven (2.0%) resulted in minor bleeding with no transfusion, three (0.8%) resulted in significant pain, one (0.3%) resulted in unplanned admission to hospital for observation, and one (0.3%) resulted in a pneumothorax [11].

In our patient, the large perforation of the tumor surface and left colon were probably not related to PCNB, but rather to the spontaneous perforation of the necrotic part of the tumor demonstrating the fast-growing of the mass. We also found peritoneal infiltration, spontaneous bleeding of the mass, and hemorrhagic ascites intraoperatively. Peritoneal infiltration should not justify a more limited resection [12]. The blood-stained ascites was an evident sign of preoperative tumor rupture [13]. Tumor rupture is an independent predictive factor associated with decreased OS and increased abdominal recurrences [4,14]. In the literature, the reported five-year survival rate in patients with intraoperative tumor rupture is 27%, and the three-year recurrence rate is 87% [4]. Furthermore, there is a lack of data about the role of spontaneous preoperative RPS rupture on oncological outcomes. Based on our experience, we suppose that the poor prognostic outcomes of intraoperative or preoperative rupture may be comparable. Our patient had a series of negative prognostic factors such as high-grade disease, peritoneal invasion, and tumor rupture [12]. To the best of our knowledge, this is the first case of spontaneous preoperative RPS rupture causing a hemoperitoneum to be reported in the literature. Very few cases of retroperitoneal hematoma due to RPS bleeding are reported in the literature [2,15-17]. While the retroperitoneal hematoma remains within the retroperitoneal compartment, leading to a higher risk of local recurrence, a hemoperitoneum spreads the tumor intraperitoneally, resulting in a higher risk of intrabdominal recurrence and peritoneal metastasis [18].

## Conclusions

Spontaneous RPS rupture is an extremely rare event. However, surgical techniques as previously described, which recommend compartmental surgery, should be considered even in this rare presentation. Spontaneous RPS rupture exposes the patient to peritoneal seeding and sarcomatosis and is associated with a poor prognosis.
